# Functional Importance of Mini-Puberty in Spermatogenic Stem Cell Formation

**DOI:** 10.3389/fcell.2022.907989

**Published:** 2022-04-28

**Authors:** Yuichi Shima

**Affiliations:** Division of Microscopic and Developmental Anatomy, Department of Anatomy, Kurume University School of Medicine, Fukuoka, Japan

**Keywords:** gonocyte, spermatogenic stem cell, mini-puberty, luteinizing hormone, follicle stimulating hormone, testosterone

## Abstract

Primordial germ cells nesting in the fetal testis give rise to gonocytes. The gonocytes then transform into spermatogenic stem cells (SSCs) during the neonatal period and thereafter serve as a lifetime source of spermatogenesis. Therefore, gonocyte to SSC transformation is quite an important process that supports fertility in males. During the gonocyte to SSC transformation, morphological and transcriptomic changes sequentially occur and gonocytes migrate from the center to the peripheral region of the seminiferous tubules. However, extrinsic signals which trigger the transcriptomic changes as well as the migration are not yet fully clarified. Recent studies have drawn attention to the temporal activation of the hypothalamic-pituitary-gonadal axis during the neonatal stage which occurs concurrently with SSC formation. This phenomenon is called mini-puberty, and recent studies on human cryptorchid patients as well as animal models partially support the hypothesis that mini-puberty plays pivotal roles in gonocyte-to-SSC transformation. Focusing on this point, here, we aimed to discuss the latest knowledge on the importance of mini-puberty in spermatogenesis in this review.

## 1 Introduction

Spermatozoa are the most specialized cell type in the male body and play indispensable roles in transferring genetic and epigenetic information from generation to generation. One of the features of male germ cell production is that spermatozoa are continuously produced and that spermatogenic stem cells (SSCs, also called spermatogonia) serve as the cellular source of spermatozoa through an individual’s lifetime. The process of SSC formation is unique and complex. Primordial germ cells (PGCs) migrate from the ectoderm to the gonadal primordium (called genital ridge) giving rise to gonocytes (Kluin and de Rooij, 1981) in the fetal testis. A part of the gonocytes then undergo apoptosis, whereas the remaining part migrates from the central part of the seminiferous tubules to the basement membrane. These migrated cells thereafter retain their stemness and serve as the source of all the germ cells. Morphological and transcriptomic changes during the gonocyte to SSC transformation have been extensively investigated (reviewed in Law and Oatley, 2020). However, extrinsic signals which trigger the gonocyte migration and SSC formation have not yet been identified.

Luteinizing hormone (LH) and follicle stimulating hormone (FSH) are secreted from the anterior pituitary and stimulate testicular Leydig and Sertoli cells, respectively. LH and FSH are collectively called gonadotropins, and gonadotropin synthesis and secretion are activated by gonadotropin releasing hormone from the hypothalamus. These hierarchical relationships between the endocrine organs are designated the hypothalamic-pituitary-gonadal (HPG) axis. In mammals, the HPG axis shows triphasic activation during fetal, neonatal, and pubertal periods. Among these, temporal activation of the HPG axis during the neonatal period is known as mini-puberty (Kuiri-Hänninen et al., 2014). Mini-puberty is concurrent with gonocyte to SSC transformation in the seminiferous tubules, and analyses of human cryptorchid patients strongly support the hypothesis that mini-puberty plays important roles in SSC formation. In contrast, studies using animal models demonstrate both positive and negative evidences for the functional importance of mini-puberty. In this review, we aimed to discuss the recent studies arguing the importance of mini-puberty in SSC formation.

## 2 Germline Development in Fetal and Neonatal Testis

### 2.1 Gonocyte Development in Fetal Testis

The most ancestral type of germ cells are the PGCs, the part of the epiblast cells which are destined to become a germline. In mice, PGCs experience several rounds of mitosis and then start to migrate from the primitive streak through the dorsal mesentery, finally settling in the gonads (Richardson and Lehmann, 2010). After sex determination, PGCs in the male gonad (testis) commit to male germ cell fate and form gonocytes (Kluin and de Rooij, 1981). Gonocytes are initially mitotically active but become quiescent at around E12.5–16.5 in mice (Western et al., 2008) and 20–25 weeks of gestation in humans (Hilscher and Engemann, 1992). Detailed and extensive analyses of mouse models revealed that a large part of the gonocytes (called differentiating spermatogonia) undergo differentiation and form the first wave of spermatogenesis. Meanwhile, a small part of gonocytes are destined to establish the SSC population which thereafter retains their stemness and provides the neurogenin3-positive spermatogenic progenitor pool that contributes to continuous sperm production (Yoshida et al., 2006).

### 2.2 SSC Formation in Neonatal Testis

After birth, heterogeneity of the male germline cells becomes apparent in terms of morphology and marker gene expression. Specifically, several marker proteins such as RET (Jain et al., 2004), PAX7 (Aloisio et al., 2014), GFRA1 (Oatley et al., 2007), and ID4 (Helsel et al., 2017) are expressed in a limited population, and these cells contribute to SSC establishment. In contrast, the SOHLH1 expressing cell population is programmed to differentiate and form the first wave of spermatogenesis (Ballow et al., 2006). These findings have been certified by recently performed single cell RNA-sequencing of the murine or human neonatal testis which enabled not only detailed clustering of germ cells but also clarified the trajectories of gonocyte to SSC transformation (Hermann et al., 2018; Law et al., 2019; Sohni et al., 2019; Tan et al., 2020). In parallel to the transcriptomic changes, gonocyte start to migrate from the luminal side to the basal side of the seminiferous tubule (McGuinness and Orth, 1992). The germ cells migrate and attach to the basement membrane and exhibit flattened morphology which is clearly distinct from the round-shaped gonocytes (Orwig et al., 2002). Although detailed molecular mechanisms regulating the migration is not yet clarified, platelet-derived growth factor and Notch signaling are thought to have some influence over the migration (Basciani et al., 2008; Garcia et al., 2013). Additionally, histological studies clarified that migrating gonocytes show increased contact with Sertoli cells (Clermont and Perey, 1957), suggesting an important role of Sertoli cells in regulating the migration of the gonocytes. Gonocyte-to-SSC transformation occurs soon after birth (2–6 days) in mice (Law and Oatley, 2020) and 3–6 months in humans (Hutson et al., 2013). A considerable number of gonocytes fail to migrate and undergo apoptosis (Orwig et al., 2002), and the unmigrated cells often serve as the source of germ cell tumor formation in cryptorchid testes in humans (Tien et al., 2020).

## 3 HPG Axis Activation During the Neonatal Stage

In humans, it has been reported that serum gonadotropin and testosterone levels show a transient surge during neonatal stages (Winter et al., 1976; Forest, 1979). Recent technical advances certified the above observations and clarified that serum gonadotropin levels peak up until 3 months and decrease to basal level at 6–9  months in humans (Kuiri-Hänninen et al., 2014). This is called mini-puberty, and the same phenomenon is also observed in mice during the first week after birth (Li et al., 2017). Several evidences support the notion that mini-puberty plays important roles for the subsequent development of male reproductive tissues such as penile growth, prostatic activity (Boas et al., 2006; Kuiri-Hänninen et al., 2011), and male-specific behavior (Lamminmaki et al., 2012).

## 4 Importance of Mini-Puberty on SSC Formation

### 4.1 Observation of Cryptorchid Patients

Cryptorchidism, also called undescended testis, is a pathological condition in which testes are not descended to the scrotum and stay at the inguinal or abdominal region. Recent technical advances on peripheral gonadotropin and testosterone measurements revealed that cryptorchidism is associated with lowered testosterone levels and is often accompanied by hypogonadotropic hypogonadism (Hadziselimović et al., 1986; Rodprasert et al., 2020). Moreover, histological observation of cryptorchid testes revealed that gonocyte migration and SSC formation was disturbed (Huff et al., 1991). These observations strongly support the hypothesis that mini-puberty plays important roles in gonocyte-to-SSC transformation (Hadziselimovic and Hoecht, 2008). Moreover, gonadotropin releasing hormone analogue treatment has reportedly improved fertility of cryptorchid patients, supporting the importance of mini-puberty in gonocyte-to-SSC transformation (Hadziselimovic and Hoecht, 2008), although there is an adverse opinion on this treatment from the viewpoint of efficacy and side effects (Thorsson et al., 2007).

### 4.2 Possible Molecular Link Between Mini-Puberty and SSC Formation

As noted above, mini-puberty was initially identified in humans, and accumulated data strongly suggests that mini-puberty plays pivotal roles in the development of reproductive functions in humans. As such, several animal models have been used to clarify the mechanistic connection between mini-puberty and SSC formation.

#### 4.2.1 Androgen

Considering the functional importance of testosterone in male reproductive function, it was conceived that testosterone also regulates the gonocyte migration and SSC formation. To support this hypothesis, it was reported that gonocyte migration is partially inhibited in complete androgen insensitivity syndrome patients (Hadziselimovic and Huff, 2002). However, a more recent and extensive study exhibited contradictory data that gonocyte migration and SSC formation are normal in 30 androgen insensitivity syndrome patients (Su et al., 2014). In the case of mice, androgen receptor (*Ar*) knockout mice showed normal gonocyte migration from E17 to P10, denying any influence of the androgen signal on gonocyte to SSC transformation (Li et al., 2015). In summary, it seems likely that testosterone is not an essential factor in SSC formation.

#### 4.2.2 FSH

It is widely accepted that FSH stimulate Sertoli cells to support spermatogenesis. However, both *Fshb* and *Fshr* knockout male mice showed reduced testis size but normal spermatogenesis, suggesting that FSH alone plays only a minor role in SSC formation (Kumar et al., 1997; Dierich et al., 1998; Abel et al., 2000). To support this hypothesis, men with the inactivating *FSHR* mutation showed reduced testis size and subfertility (Tapanainen et al., 1997). However, the *in vitro* culture of rat testis suggested the possibility that FSH regulates SSC formation in combination with follistatin (Meehan et al., 2000).

## 5 Discussion

As noted above, positive results were mainly provided from studies of cryptorchidism in humans. However, there is a considerable number of contradictory results even in studies on humans, preventing any concrete conclusion. Similarly, the results of animal studies are also confusing, and the consensus on the importance of mini-puberty has not yet been firmly achieved. In addition, we should also consider the interspecies differences in the role of the HPG axis on reproductive function. Taken together, the functional importance of mini-puberty is still under debate. In future studies, the impact of mini-puberty on SSC formation should be clarified at the molecular level, and such studies are expected to clarify the pathogenesis of male infertility and expand the possibilities of its treatment.

## Abbreviations

FSH, follicle stimulating hormone; HPG axis, hypothalamic-pituitary-gonadal axis; LH, luteinizing hormone; PGC, primordial germ cell; SSC, spermatogenic stem cell.
